# Comparison of early growth and development in very preterm children in the Netherlands between the 1980s and 2000s

**DOI:** 10.1186/s12887-026-06857-9

**Published:** 2026-04-29

**Authors:** Réka E. Sexty, Paula van Dommelen, Arend F. Bos, Sylvia van der Pal, Dieter Wolke, Sijmen A. Reijneveld, Peter Bartmann

**Affiliations:** 1 Department of Psychology, Health Psychology Unit, University of Graz, Graz, Austria; 2 University Hospital Bonn, Children’s Hospital, Bonn, Germany; 3 Department of Child Health, TNO, Leiden, the Netherlands; 4 Division of Neonatology, Beatrix Children’s Hospital, University Medical Center Groningen, University of Groningen, Groningen, the Netherlands; 5 Department of Psychology, University of Warwick, Coventry, UK; 6 Department of Health Sciences, University Medical Center Groningen, University of Groningen, Groningen, the Netherlands

**Keywords:** Growth, Neurodevelopment, Very preterm infant, POPS, LOLLIPOP, RECAP preterm

## Abstract

**Background:**

Since 1990, the survival rate of very preterm (VP) infants (born < 32 weeks of gestation) has significantly improved due to advancements in neonatal care practices. This study aimed to investigate whether these medical advances have translated into better growth and neurodevelopmental outcomes during the early life of VP children.

**Methods:**

A comparative analysis was performed using data from two community-based studies: POPS (1983) and LOLLIPOP (2002/2003). A total of 1,294 very preterm (VP) infants provided data on height, weight, and head circumference during the first two years of life, as well as fine motor skills, gross motor skills, and communication milestones at 24 months of age. Rigorous harmonization procedures were applied to ensure comparability, including the standardization of growth metrics and consistent definitions for neurodevelopmental milestones across cohorts. Analyses were adjusted for higher parental education, severe intraventricular haemorrhage (IVH) and extended neonatal hospital stay to uncover if improvements reduce the differences in growth and neurodevelopment.

**Results:**

The LOLLIPOP cohort showed better height and weight growth trajectories within the first 24 months of life, even after controlling for sociodemographic and neonatal factors. Differences in head circumference trajectories were significant in unadjusted analyses but lost significance when adjusted for higher parental education, severe IVH, and extended hospital stays. Neurodevelopmental milestones, particularly gross and fine motor skills (e.g., “imitates others” and “squats or bends to pick things up”), improved in the LOLLIPOP cohort but were attenuated after adjustment. Substantial delays in communication (e.g., “says sentences of two words”) and gross motor milestones (e.g., “walks well alone”) were observed across both cohorts compared to the norms derived from full-term children.

**Conclusion:**

VP infants born in the 2000s exhibited modest improvements in growth outcomes compared to those born in the 1980s. Reductions in neurodevelopmental impairments for the VP infants born in the 2000s are associated with higher parental education levels and reduced neonatal complications. Despite these advancements, persistent delays in cognitive and motor development underscore the necessity for early and targeted interventions to support VP children effectively.

**Supplementary Information:**

The online version contains supplementary material available at 10.1186/s12887-026-06857-9.

## Background

Very preterm (VP) birth (< 32 weeks of gestation) is associated with a delay in growth [1–3] and development [4–7] during childhood. The survival rate of VP neonates, especially those at the limits of viability, has increased dramatically over the past few decades [8]. Consequently, several new therapies have been developed for obstetrical and neonatal care practices since the early 1990s, including the implementation of antenatal corticosteroids for induction of foetal lung maturation, nasal continuous positive airway pressure, high-frequency ventilation, and intratracheal application of surfactant [9]. There is considerable scientific, practical and economic interest in determining whether these expanded neonatal care practices have led to better health outcomes and improved development in children born VP across various countries.

Within-country investigations, such as EpiCure and EPIPAGE studies, which analysed developmental data from different eras, have observed improvements in the early years of children born VP. These studies found a higher rate of survival without disabilities at 2–3 years of age in cohorts born in the 2000s compared to earlier cohorts [10–12]⁠. However, the proportion of severe developmental problems at school age has not changed over time [13–15].

Given the differences in care protocols, it is valuable to compare growth and developmental changes within a single country. The authors of the present study previously conducted a comparison of neonatal morbidity and care practices in the Netherlands between the 1980s and 2000s [16]. By harmonizing perinatal characteristics, neonatal morbidities and care practices between the two cohorts, they revealed a decrease in prevalence of severe intraventricular haemorrhage (IVH) and sepsis, as well as reduced length of NICU and hospital stays, alongside an increase in the use of continuous positive airway pressure, mechanical ventilation and caffeine therapy by 2003.

However, it remains unclear whether changes in neonatal care practices over time have had a positive impact on growth and development in Dutch children born VP. It is also uncertain how these outcomes relate to other relevant factors such as parental education⁠, IVH grade III-IV and length of newborn hospital stay (as a surrogate parameter for disease severity) or differences in socioeconomic status [17].

The aim of this study is to compare early growth and neurodevelopment in children born VP in the 1980s and 2000s, using national-level data from the Netherlands. Two well-defined, community-based Dutch cohorts of VP infants – the Project On Preterm and Small for Gestational Age infants (POPS) and the Longitudinal Preterm Outcome Project (LOLLIPOP), will be used. Both cohorts share similar inclusion criteria and partly employ identical measures of growth and neurodevelopment. Specifically, we were interested whether there are differences in height, weight, and head circumference trajectories in the first two years of life, as well as the attainment of key neurodevelopmental milestones at 2 years of corrected age, between the two eras. We aimed to examine how sociodemographic and neonatal factors contribute to the cohort differences.

## Methods

### Study populations

This study is a longitudinal follow-up of two Dutch cohorts of children aged 0–2 years who were born VP. POPS cohort is a prospective population-based cohort consisting of 1,338 VP and/or very low birth weight (VLBW) infants who were live-born in 1983 in the Netherlands, including 94% of the whole eligible population of the country.

The LOLLIPOP cohort is a community-based cohort including both preterm and full-term infants, born in 2002 and 2003. All preterm children who attended the last Preventive Child Health Care (PCHC) visit at ages 43–49 months were included, representing approximately 25% of this age group population in the country. 1,983 children participated in LOLLIPOP. A more detailed description of both cohorts is available in van der Pal-de Bruin et al. 2015 [18] and Kerstjens et al. 2011 [19], respectively.

## Harmonization of the two cohorts^1^

**Fig. 1:**
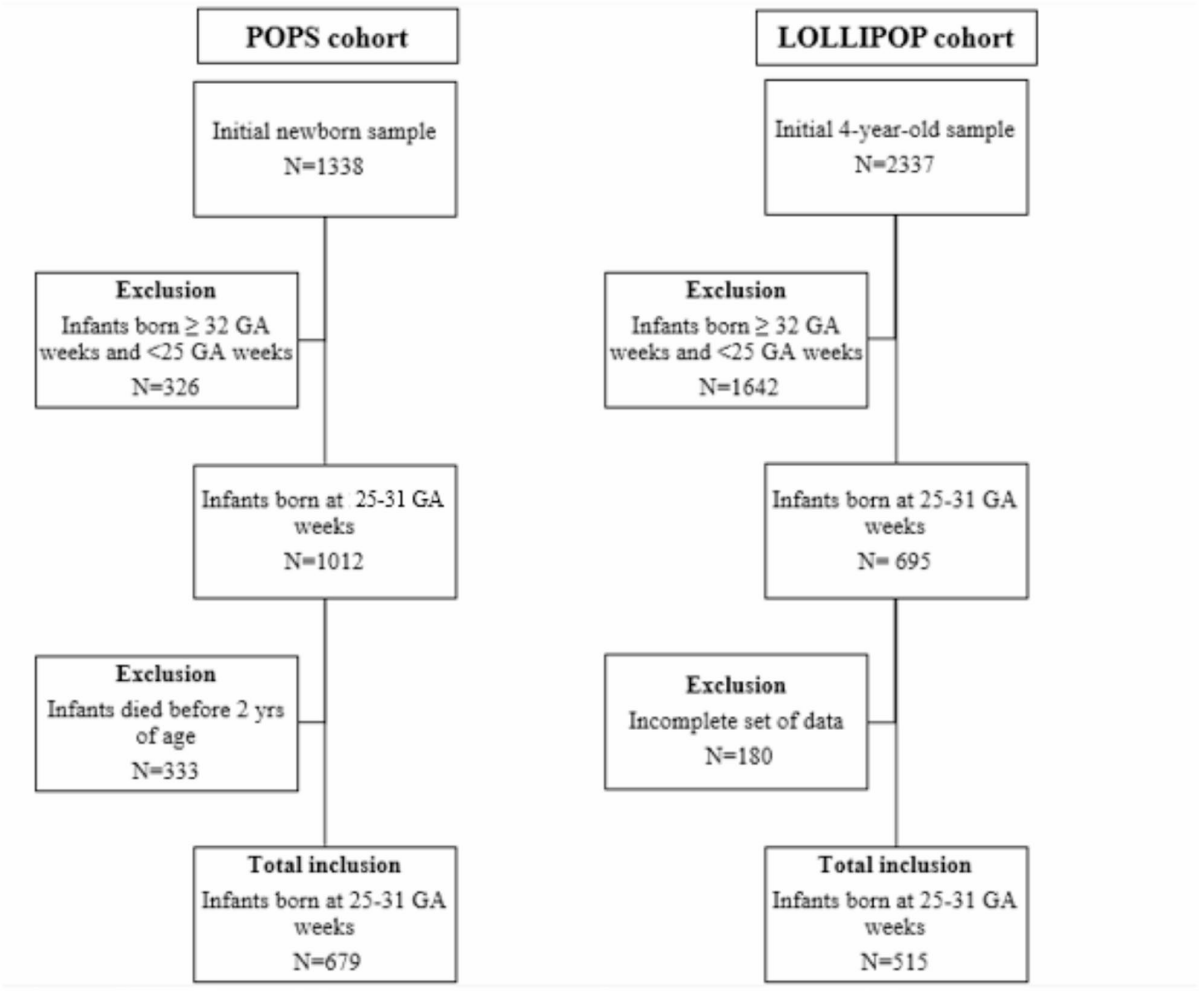
Selection of POPS and LOLLIPOP participants for the present study

The POPS cohort included infants born VP (< 32 weeks gestational age [GA]) or VLBW (< 1500 g), while LOLLIPOP only included infants born < 32 weeks GA. For the present analysis, harmonized inclusion criteria were applied to both cohorts to ensure comparability of the datasets: (1) GA at birth: 25–31 weeks, (2) absence of severe congenital malformations (3) survival to 2 years of age, (4) complete perinatal data and at least one measurement of growth^2^ or development^3^. Figure 1 provides an overview how participants of both cohorts were selected. The final data set comprised *N* = 679 infants from the POPS cohort and *N* = 515 from the LOLLIPOP cohort.

In the POPS cohort, children who died before 24 months of age were more likely being born at lower GA, lower birth weight (BW), lower APGAR scores, in breech presentation and without caesarean section, and were often multiples, compared to survivors (data not shown). Deceased children more frequently had severe IVH and were more likely to have received mechanical ventilation, but less likely to have received continuous positive airway pressure (data not shown).

In the LOLLIPOP cohort, exclusions were mainly due to missing neonatal data and lack of follow-up participation. Neonatal data were unavailable for 148 children because parents did not grant permission for retrospective analysis of perinatal data or because neonatal records could not be retrieved. Additionally, 34 children did not participate in any growth or developmental assessments and were therefore excluded. Excluded children did not significantly differ from included participants in small-for-gestational-age status or multiple birth (data not shown). However, the included group comprised slightly more girls (49.8% vs. 42.2% χ² [1] = 5.11, *p* < 0.05), infants born at more advanced GA (29.26 ± 1.60 vs. 28.96 ± 1.62, t(806) = 279, *p* < 0.01 and birth weight (1300 ± 365 vs. 1243 ± 341, t(834) = 2.44, *p* < 0.05), and less mothers reporting previous pregnancies (40.9% vs. 26.2%, χ² [1] = 11.12, *p* < 0.05).

The number of children who participated in at least one height, weight, head circumference or developmental score measurement, as well as the total number of measurements in both cohorts, are presented in Supplementary Table 1.

## Measures

### Data collection

POPS is a prospective cohort study. Perinatal data up to discharge were collected in participating hospitals, and follow-up assessments are still ongoing into adulthood. Data were recorded on pre-coded forms for neonatal morbidity and parental physical and social information. After discharge, POPS participants visited a paediatrician at the hospital at 3, 6, 12 and 24 months of corrected age for prematurity (calculated from the original expected due date). The paediatrician completed a medical examination form that included growth data and administered the Dutch Developmental Instrument (DDI).

In the LOLLIPOP cohort, children were recruited at Preventive Child Healthcare Centers (PCHC) during routine health check-ups at 43–49 months of age. Growth and developmental milestone data for earlier ages were extracted retrospectively from records of routine child health visits where growth data was recorded, and milestones were actively observed by the PCHC physicians at standardized time-points between 1 and 24 months of age according to local protocols.

Perinatal and neonatal data were collected from various sources, including a general parental questionnaire, birth registers, PCHC records, and medical records of both mother and child, allowing for cross-checking of information.

### Perinatal data

The authors of this study harmonized perinatal data between cohorts for the analysis following harmonization guidelines [20–22]. A more detailed description of the harmonization process is available in Sexty et al. 2023 [16].For the present study, we selected perinatal data proposed to have predictive value for growth and neurodevelopmental outcomes. Parental socioeconomic status has been shown to impact neurodevelopment and cognitive abilities of children from birth into early childhood, especially in premature settings [23, 24]. Low maternal education was found to be a particularly strong predictor for language delay at 2 years of age in children born VP, more so than various neonatal morbidities [25]. Additionally, male sex, low GA, low BW, and belonging to an ethnic minority have been identified as the strongest predictors of cognitive impairments in early childhood [26]. Severe IVH and periventricular leukomalacia are known to predict cerebral palsy [27] and partially poor cognitive outcomes in the early years [26].

Based on the scientific evidence and the result of the harmonization of perinatal data, we selected GA, sex, parental education, severe IVH and length of hospital stay after birth as potential perinatal covariates. Parental education was treated as a potential confounder, whereas IVH and length of hospital stay were considered postnatal characteristics that may partly mediate cohort differences. The length of hospital stay was used as a proxy for the overall neonatal course, acknowledging that hospital stay may also reflect institutional discharge policies and care practices.

### Growth and developmental measures

Growth data included length/height and weight measurements from 0 to 27 months, as well as head circumference from 0 to 15 months. Height standard deviation score (HSDS), Weight standard deviation score (WSDS) and head circumference standard deviation score (HCSDS) were calculated based on GA-specific growth references [2]. These growth references are based on calendar age. Therefore, additional correction for prematurity was not applied in the growth trajectory analyses.

Dutch preventive child healthcare centres (PCHC) use the DDI, a modification of the Gesell test, to assess the development of children from birth to 4 years of age [28]⁠. The DDI consists of 75 age-specific developmental milestones across three main domains: (1) fine motor skills, adaptive/personal/social behaviour milestones; (2) communication; and (3) gross motor skills [28]⁠. The Developmental Score (D-score) is derived from an algorithm that summarizes the pass/fail scores of developmental milestones into a single aggregate score reflecting global development [29]. It is a validated, unified indicator for early life development [30]. The advantage of using the D-score as a single measure is that it is on an interval scale, enabling the ranking of individuals, groups, or populations from low to high ability.

In this study, the D-score was determined at the 24-months visit. Since the D-score increases with age, adjustment using age-specific reference data (similar to growth charts) [30]. Adjusted D-scores for age (DAZ) were therefore calculated. Because gestational-age-specific reference based on chronological age were not available, corrected age was used for these analyses.

Additionally, we identified five DDI milestones administered at 24 months that were assessed in both cohorts. To ensure comparability, we included only these five milestone items that were identical in wording across both cohorts and had sufficient data for analysis (available for more than 90% of participants in each cohort). The selected milestones were analysed individually—beyond the overall D-score—as they provide specific information on distinct facets of early development (e.g., motor, language, and social domains) and represent critical indicators for understanding neurodevelopment at this age [31].

### Statistical analysis

Chi-square tests (%), Mann-Whitney-Wilcoxon tests (for interquartile ranges), and Student’s t-tests (for means and standard deviation (SD)) were applied to test differences in characteristics between the POPS and LOLLIPOP cohorts, as well as between included and excluded participants within each cohort.

To describe and compare growth outcomes between the cohorts in the first 24 months of life, we performed two types of growth outcome analyses: one with interval data (4–24 months) and another with cut-off points applied at 24 months of age.

**Fig. 2:**
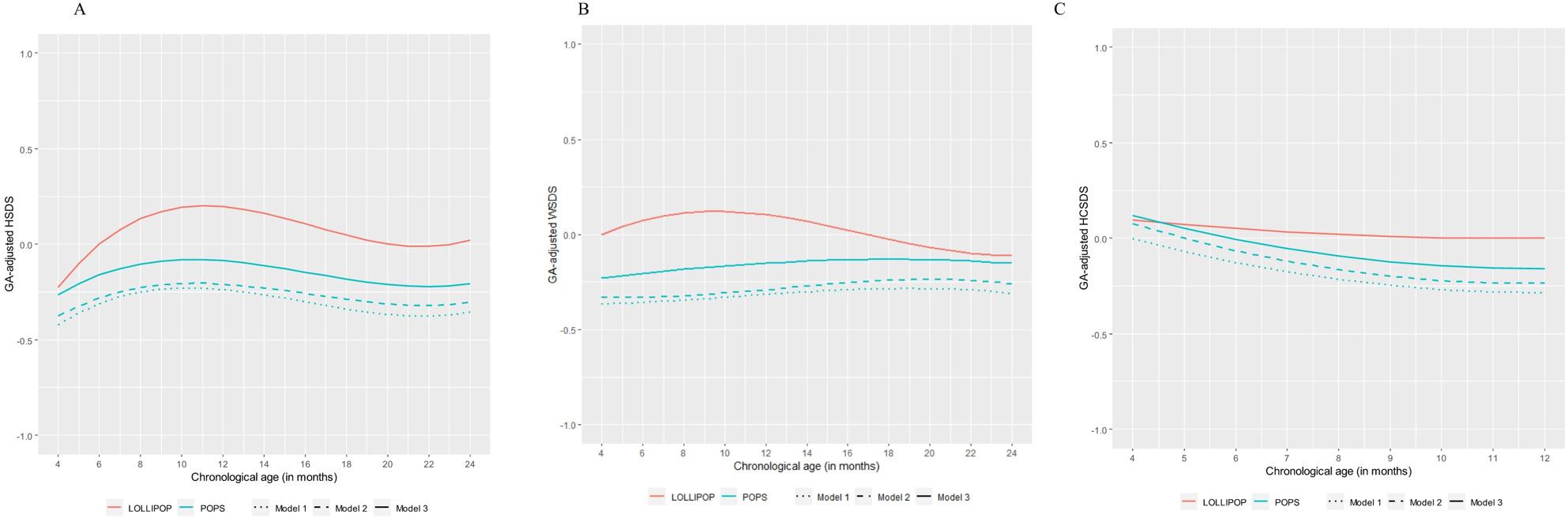
GA-adjusted z-score trajectories of height (**A**) weight (**B**), and head circumference (**C**) for POPS and LOLLIPOP

First, three linear mixed-effects models were fitted for each growth outcome: HSDS, WSDS and HCSDS. For model fitting, we selected children with a chronological age of 4–24 months for height and weight, and 4–12 months for head circumference. The first model included cohort (POPS vs. LOLLIPOP) and a cubic polynomial of chronological age for height and weight, and a quadratic polynomial for head circumference, along with their interaction as independent variables. The second model included parental education (high vs. medium/low) in addition to the first model’s variables. The third model additionally included IVH (grades 3–4 vs. no IVH or grades 1–2) and length of hospital stay (with natural logarithms applied for normality) as independent variables. All models applied a random slope (and intercept) for the effect of age (with the appropriate polynomial for each outcome) for each child. For visualizing the models (Figs. 2A-C), we used chronological ages 4–24 months for height and weight, and 4–12 months for head circumference, as these periods had the most data available. To visualize the difference in outcomes between POPS and LOLLIPOP, we estimated the predicted outcomes from each regression model, successively adjusting for potential confounders and postnatal characteristics. Predicted outcomes were calculated under the assumption that both cohorts had the same distribution of the included covariates.

Second, we analysed differences in the proportion of children with HSDS, WSDS, or HCSDS ≥ -2SD using logistic regression analyses at specific time points. We used 24–25 months of chronological age for height and weight, and 11–13 months of chronological age for head circumference, since standardized reference data are available only up to around 12 months of age. At this point, rates of overweight were also calculated based on the extended International Obesity Task Force (IOTF) cut-off values [32]. The first model included cohort (POPS vs. LOLLIPOP). The second model added corrected age at assessment and GA. The third model further included parental education (high vs. medium/low). The fourth model included IVH (grades 3–4 vs. no IVH or grades 1–2) and length of newborn hospital stay (with natural logarithms applied for normality) as independent variables.

Developmental data using DAZ categorized into low (defined as <-2 SD) versus medium/high (≥-2 SD), were compared at one timepoint (24–25 months of corrected age) between the cohorts. Four logistic regression models were applied with the same structure as for the HSDS, WSDS and HCSDS outcomes.

A p-value of < 0.05 (two-sided) was considered statistically significant. The statistical software package and R 3.6.2 (R Core Team, 2019) were used for the calculations.

## Results

### Characteristics of the study population

Table 1 presents the characteristics of the study population. Children in the LOLLIPOP cohort had parents with higher levels of education (high or medium), fewer cases of severe IVH (grades 3–4) and a shorter length of hospital stay after birth compared to the POPS cohort.

**Table 1:** Sociodemographic and neonatal characteristics of the study population

Parameter		POPS	LOLLIPOP	*p*-value
Total number of children		679	515	
		P50 (P25-P75)	P50 (P25-P75)	
Gestational age (weeks)		30 (28–31)	30 (28–31)	0.21^6^
		% (N)	% (N)	
Sex	Girls	46% (313)	50% (255)	0.27^7^
	Boys	54% (366)	50% (260)	
Parental educational level (ISCED)^1^	Low	38% (235)	18% (92)	**< 0.001** ^**7**^
	Medium	37% (229)	41% (210)	
	High	25% (158)	41% (207)	
Intraventricular haemorrhage (IVH)^2^	No IVH or IVH grades 1–2	91% (458)	96% (461)	**< 0.001** ^**7**^
	IVH grades 3–4	9% (47)	4% (17)	
		P50 (P25-P75)	P50 (P25-P75)	
Length of hospital stay (days)^3^		65 (50–82)	54 (43–71)	**< 0.001** ^**6**^
		Mean (SD)	Mean (SD)	
GA-adjusted birthweight SDS		-0.4 (1.0)	-0.5 (1.1)	0.24^8^
GA-adjusted birth length SDS^4^		-0.3 (1.0)	-0.5 (1.0)	0.06^8^
GA-adjusted birth head circumference SDS^5^		-0.3 (0.9)	-0.3 (0.9)	0.88^8^

*ISCED* International Standard Classification of Education

^1^*n* = 622 available for POPS, *n* = 513 available for LOLLIPOP, ^2^*n* = 505 for POPS, *n* = 482 for LOLLIPOP, ^3^*n* = 441 for LOLLIPOP, ^4^*n* = 507 for POPS, *n* = 98 for LOLLIPOP, ^5^*n* = 563 for POPS, *n* = 173 for LOLLIPOP, ^6^Mann-Whitney-Wilcoxon Test, ^7^Chi-square test, ^8^t-test

Bold values indicate statistically significant effects

### Growth outcomes in the first 24 months of life

Table 2 shows the results of the linear mixed-effect models with HSDS, WSDS, and HCSDS as outcomes. Figure 2 illustrates the trajectories of HSDS (A), WSDS (B), and HCSDS (C) by age, based on models 1, 2 and 3 (see Table 2) for the POPS and LOLLIPOP cohorts.

**Table 2:** Results of the linear mixed-effect models with height, weight, and head circumference as outcomes

	GA-adjusted HSDS (4-24 months of chronological age)			GA-adjusted WSDS (4-24 months of chronological age)			GA-adjusted HCSDS (4-12 months of chronological age)		
	Model 1	Model 2	Model 3	Model 1	Model 2	Model 3	Model 1	Model 2	Model 3
Parameter	Est. (SE)	Est. (SE)	Est. (SE)	Est. (SE)	Est. (SE)	Est. (SE)	Est. (SE)	Est. (SE)	Est. (SE)
Parameter	Est. (SE)	Est. (SE)	Est. (SE)	Est. (SE)	Est. (SE)	Est. (SE)	Est. (SE)	Est. (SE)	Est. (SE)
Intercept	-0.29 (0.04)***	-0.3 (0.11)**	3.39 (0.33)***	-0.33 (0.04)***	-0.39 (0.1)***	3.61 (0.32)***	-0.18 (0.04) ***	-0.69 (0.11) ***	2.47 (0.36) ***
Poly (age^a^, 3) 1	0.20 (1.05)	0.55 (1.09)	0.02 (1.22)	1.12 (0.99)	1.6 (1.02)	1.51 (1.12)	-4.71 (0.76) ***	-5.04 (0.77) ***	-4.5 (0.89) ***
Poly (age^a^, 3) 2	-2.04 (0.78)**	-1.98 (0.81)*	-2.26 (0.92)*	-1.63 (0.7)*	-1.53 (0.73)*	-1.79 (0.83)*	2.96 (0.65) ***	3.48 (0.67) ***	3.08 (0.76) ***
Poly (age^a^, 3) 3	3.46 (0.84)***	2.87 (0.87)***	3.16 (1)**	-0.57 (0.65)	-1.05 (0.67)	-0.09 (0.77)			
Cohort (LOLLIPOP vs. POPS)	0.37 (0.05)***	0.33 (0.06)***	0.22 (0.06)***	0.40 (0.05)***	0.37 (0.06)***	0.22 (0.06)***	0.20 (0.06)***	0.14 (0.06)*	0.09 (0.06)
Poly (age^a^, 3) 1: cohort	3.35 (1.5)*	3.02 (1.54)	3.37 (1.67)*	-5.63 (1.45)***	-6.16 (1.48)***	-5.96 (1.58)***	3.29 (1.14)**	3.61 (1.14)**	3.01 (1.28)*
Poly (age^a^, 3) 2: cohort	-3.15 (1.08)**	-3.25 (1.11)**	-2.84 (1.23)*	-1.14 (1.01)	-1.22 (1.03)	-0.92 (1.16)	-2.03 (0.92)*	-2.51 (0.94)**	-1.97 (1.04)
Poly (age^a^, 3) 3: cohort	3.00 (1.02)**	3.58 (1.05)***	3.01 (1.19)*	3.43 (0.85)***	3.78 (0.87)***	2.75 (0.96)**			
Parental education (medium/high vs. low)		0.02 (0.06)	-0.04 (0.07)		0.05 (0.06)	-0.003 (0.06)		0.31 (0.07) ***	0.19 (0.07)**
IVH (grades 3–4 versus lower or no IVH)			0.33 (0.12)**			0.19 (0.12)			0.11 (0.13)
natural logarithm of length of hospital stay (days)			-0.87 (0.07)***			-0.94 (0.07)***			-0.72 (0.08) ***

Model 1: cohort + polynomial of age (*N* = 679 within POPS and *N* = 519 within LOLLIPOP)

Model 2: cohort + polynomial of age, parental education (*N* = 622 within POPS and *N* = 513 within LOLLIPOP)

Model 3: cohort + polynomial of age, parental education, IVH, natural logarithm of length of hospital stay (*N* = 458 within POPS and *N* = 420 within LOLLIPOP)

^a﻿^age= chronological age for GA-adjusted HSDS (Height Standard Deviation Score), WSDS (Weight Standard Deviation Score), HCSDS (Head Circumference Standard Deviation Score)

Bold values indicate statistically significant effects. Asterisks denote significance levels (*p < 0.05, **p < 0.01, ***p<0.001)

Linear mixed-effect models revealed significant differences between the cohorts for HSDS and WSDS, with LOLLIPOP children showing higher values between 4 and 24 months of age. This difference remained constant when the covariates polynomial age and parental education were included in the models. However, the effect size of the cohort difference decreased when severe IVH and length of hospital stay were additionally entered into the model. Regarding HCSDS, LOLLIPOP children showed a better trajectory outcome between 4 and 12 months of age. Adjusting for polynomial age and parental education, this result remained. However, the difference was no longer significant after adjusting additionally for severe IVH and longer length of neonatal hospital stay.

The proportion of children with a HSDS ≥ -2SD or HCSDS ≥ -2SD was significantly higher in LOLLIPOP compared to POPS (Table 3). However, these differences decreased and were no longer significant when adjusted for GA, corrected age at assessment, and higher parental education, as well as for severe IVH and longer length of newborn hospital stay. Rates of overweight did not differ significantly between the cohorts.

**Table 3:** Differences in proportion of standardized z-score categories above − 2SD of growth in POPS and LOLLIPOP

Outcome	POPS % (*n*/*N*)	LOLLIPOP % (*n*/*N*)	OR (95% CI)	Adj^1^ OR (95% CI)	Adj^2^ OR (95% CI)	Adj^3^ OR (95% CI)
HSDS ≥-2 (vs. <-2)^4^	92.7 (166/179)	98.6 (275/279)	5.4 (1.7–16.8)**	4.1 (1.1–14.8)*	2.7 (0.7–10.4)	1.4 (0.4–5.7)
WSDS ≥-2 (vs. <-2)^4^	93.0 (185/199)	96.8 (272/281)	2.3 (1.0-5.4)	2.6 (1.0-7.2)	2.1 (0.7–6.4)	1.3 (0.4–4.9)
HC SDS ≥-2 (vs. <-2)^5^	93.5 (130/139)	97.5 (390/400)	2.7 (1.1–6.8)*	3.4 (0.8–14.6)	3.6 (0.8–16.4)	2.7 (0.6–12.3)
% Overweight^4^	3.9 (7/179)	2.3 (6/264)	0.6 (0.2–1.7)	0.7 (0.2–2.4)	0.6 (02-2.4)	0.6 (1.2–2.8)

^1^Adjusted for GA (weeks) and corrected age,

^2^Adjusted for GA (weeks), corrected age and parental education,

^3^Adjusted for GA (weeks), corrected age and parental education, IVH, natural logarithm of length of hospital stay

^4^Around 2y chronological age

^5^Around 1y chronological age

Bold values indicate statistically significant effects. Asterisks denote significance levels (*p < 0.05, **p < 0.01, ***p<0.001)

### Developmental outcomes at 24 months of age

We compared cohort differences in the proportion of children with a DAZ ≥ − 2 SD and in passing five specific milestones (see Table 4). At around 24 months of corrected age, a higher proportion of children in the LOLLIPOP cohort had a DAZ ≥ − 2 SD compared to POPS. This difference remained significant in Model 1, adjusted for gestational and corrected age, but was no longer significant in Model 2 (additionally adjusted for parental education) or Model 3 (further adjusted for severe IVH and length of hospital stay).

**Table 4:** Differences in proportion of standardized z-score above − 2SD of developmental outcomes and in pass rate of milestones between POPS and LOLLIPOP cohorts

Outcome	POPS % pass or yes (*n*/*N*)	LOLLIPOP % pass or yes (*n*/*N*)	OR (95% CI)	Adj 1 OR (95% CI)	Adj 2 OR (95% CI)	Adj 3 OR (95% CI)
DAZ ≥-2 (vs. <-2)^4^	93.2 (533/572)	98.4 (367/373)	4.5 (1.9–10.7)***	4.3 (1.6–11.3)**	2.6 (0.9–7.2)	1.1 (0.3–3.6)
Builds tower of three cubes (F)	90.8 (504/555)	88.8 (270/304)	0.8 (0.5–1.3)	1.2 (0.7–2.1)	0.8 (0.4–1.6)	0.4 (0.2–0.9)*
Imitates others (F)	94.8 (525/554)	97.9 (329/336)	2.6 (1.1-6.0)*	2.9 (1.1–7.7)*	2.3 (0.8–6.7)	0.9 (0.2–3.4)
Says ‘sentences’ of two words (C)	75.7 (429/567)	73.6 (257/349)	0.9 (0.7–1.2)	1.2 (0.8–1.7)	0.9 (0.6–1.4)	0.8 (0.5–1.3)
Squats or bends to pick things up (G)	89.1 (480/539)	96.0 (310/323)	2.9 (1.6–5.4)***	4.3 (2.1–8.7)***	3.7 (1.7–7.7)***	2.0 (0.8–4.6)
Walks well alone (G)	90.0 (513/570)	91.6 (318/347)	1.2 (0.8–1.9)	1.3 (0.8–2.4)	1.0 (0.6–1.9)	0.6 (0.3–1.2)

^1^Adjusted for GA (weeks) and corrected age

^2^Adjusted for GA (weeks), corrected age and parental education

^3^Adjusted for GA (weeks), corrected age and parental education, IVH, natural logarithm of length of hospital stay

^4^around 2y corrected age

^*^p < 0.5, ^**^p < 0.01, ^***^p < 0.001, Developmental domains: C, communication; F, fine motor activity, adaptive behavior, and personal/social behavior; G, gross motor activity

Children in the POPS cohort were significantly less likely to pass the milestones *“imitates others”* and *“squats or bends to pick things up”*. These differences persisted after adjusting for GA and corrected age but disappeared after additional adjustment for better parental education as well as for severe IVH and longer hospital stay. The attenuation of the cohort differences was mainly driven by severe IVH and longer hospital stay.

For the milestone *“builds tower of three cubes”*, the difference became significant in the opposite direction in Model 3, indicating greater difficulty among children in the LOLLIPOP cohort after accounting for severe IVH and longer hospital stay. However, as five outcomes were tested simultaneously, correction for multiple comparisons using the Holm method rendered this association non-significant.

## Discussion

This study demonstrates clear improvements in growth among very preterm infants born in the 2000s compared with those born in the 1980s. Height and weight trajectories during the first two years of life were more favorable in the later cohort, even after accounting for sociodemographic and neonatal differences. In contrast, improvements in neurodevelopmental outcomes were limited.

At 24 months of corrected age, the proportion of children with growth restriction or developmental delay was lower in the LOLLIPOP cohort, but these differences diminished after adjustment for parental education and neonatal morbidity. Because lower parental education and severe IVH lie on the causal pathway leading to poorer growth and neurodevelopmental outcomes, adjusting for these factors may have partially controlled for genuine improvements in the LOLLIPOP cohort.

We observed improved growth trajectories (length and weight) in VP infants from the LOLLIPOP cohort, consistent with the findings of Ruys et al. 2019 [33], who compared growth outcomes in the POPS cohorts with data from another Dutch cohort from the 2000s, the STEP cohort. These authors also reported a higher height SDS in the 2000s cohort at 24 months of corrected age. Schönbeck et al. [34] reported a continuing upward trend in height growth among Dutch children from the general population between 1955 and 1997, with particularly pronounced gains after 4 years of age and in final height. However, no clear secular growth trend was indicated in the first two years of life, this ongoing secular increase – also during the 1980–1997 period – may partly explain why VP children born in the 2000s were taller at 24 months of corrected age than those born in the 1980s. In another study [35] including both preterm and full-term children, similar trends were observed in the control (full-term) group, suggesting that part of the improvement in preterm growth reflects broader secular changes rather than cohort-specific medical factors. In the present study, only the LOLLIPOP cohort included data from a full-term reference group, which prevented us from controlling for possible secular trends in our analyses. This uncertainty should therefore be considered a limitation of the present study, and any observed growth improvements should be interpreted with caution.

In contrast to height trends, national registry data showed similar median body mass index (BMI) values for 2-year-old children in 1980 and 1997 [36], which is in line with unchanged overweight rates between POPS and LOLLIPOP.

The improved growth outcomes of VP infants from the 1980s to the 2000s can be attributed to changes in the nutrition practices in neonatal care. Early parenteral nutrition has generally been associated with better growth outcomes of preterm infants throughout eras [37]. Protein-reach nutrition, as opposed to fat-rich diet, led to better growth outcomes in preterm infants, but did not seem to affect neurodevelopment in early childhood [38]. Breast milk has also been already recommended for preterm infants since the 1990s to promote improved health [39]. Although formal international guidelines on protein intake through parenteral nutrition (e.g., ESPGHAN/ESPEN) were published only later [40], increasing emphasis on early protein provision was already evident in earlier studies, including initiation of enteral feeding on day 1 and intravenous amino acid administration within the first 24 h. Such practices were commonly implemented in Dutch NICUs in the early 2000s, from which the LOLLIPOP cohort originated.

We found significant differences in HC trajectories up to 12 months of age between the 1980s and the 2000s cohorts, consistent with the findings of [33]. 2.5% of LOLLIPOP infants showed severe HC retardation at 12 months, which is comparable to the proportion reported by Wright & Emond, 2015 [41]. In contrast, 6.5% of POPS children had HC impairments around 12 months of age. However, the significant differences in HC trajectories in the first 24 months of life and in HC retardation at 12 months of age between cohorts did not persist after adjusting for better parental education, severe IVH and longer length of hospital stay. This result confirms that neonatal morbidities, as severe IVH, can influence head growth in the first months and years of life [42, 43]. We can also assume that VP infants’ longer hospital stay after birth is associated with other severe neonatal morbidities such as bronchopulmonary dysplasia (BPD) or necrotizing enterocolitis which may inhibit normal head growth in the early years of life [44–46]. In our interpretation, length of hospital stay was considered as a proxy for early neonatal complications. However, given potential differences in clinical management and discharge policies across decades, its interpretation as a morbidity indicator requires caution.

Furthermore, better parental education appears to contribute to less growth impairment in the LOLLIPOP cohort. Higher maternal education has been associated with longer breastfeeding periods and better neonatal feeding practices [47, 48]. Breastfeeding is known to improve short-term and long-term health conditions and developmental outcomes in both full-term and preterm children [49, 50]. In this sense, higher educational levels may indirectly support better growth outcomes in VP infants through increased breastfeeding.

Recent studies have reported improvements in neurodevelopment among VP infants during the first years of life, with a higher proportion of children free from neurodevelopmental impairment [8, 51], which is consistent with our unadjusted findings. However, other studies have concluded that the prevalence of severe impairments across various neurodevelopmental domains has remained largely unchanged over time [13, 15, 52]. A recent meta-analysis [53] suggests that advances in neonatal and family-centred care produce short-term benefits—up to the first two years—for cognitive outcomes, but these improvements tend to diminish at later ages.

Differences in neurodevelopmental outcomes between the POPS and LOLLIPOP cohorts at 24 months of corrected age disappeared after adjustment for parental education, severe IVH and for the proxy indicator of neonatal morbidity, length of hospital stay. This finding is in line with recent reviews concluding no neurodevelopmental improvement in preterm born children over decades [54, 55]. The higher proportion of highly educated parents and the reduction in severe neonatal complications in the 2000s likely contributed to improved fine and gross motor functions, such as *“imitates others”* and *“squats or bends to pick things up”.* Nevertheless, these advantages did not translate into consistent overall improvements in neurodevelopmental outcomes.

Cheong et al. [52] likewise reported significant associations between neurodevelopmental outcomes at 2 years and both neonatal characteristics (e.g., GA, BW) and sociodemographic factors (e.g., maternal education). At school age, lower parental education, severe IVH, cystic periventricular leukomalacia, and neonatal surgery have been identified as major predictors of neurodevelopmental impairment [13]. Beyond advances in neonatal care — such as the introduction of surfactant therapy — and thereby improvements in neonatal morbidity, other era-related factors should also be considered. These could include sociodemographic and health behaviour changes over time, such as increasing maternal age at childbirth, higher rates of primiparity, rising parental educational levels, and declining smoking prevalence [16].

While somatic growth (weight and length/height) is often considered in relation to neurodevelopment, evidence suggests that head circumference growth is more strongly associated with neurodevelopmental outcomes extending into adulthood [56]. In our cohort comparison, however, head circumference growth did not show relevant changes over time. This may explain why improved weight and length/height in the more recent cohort were not accompanied by clear improvements in developmental outcomes. In addition, observed improvements in increased height and weight trajectories should be interpreted cautiously, as they may partly reflect secular trends rather than cohort-specific effects.

Our findings contribute to the growing body of literature suggesting limited progress in neurodevelopmental outcomes among preterm children across birth eras. Previous studies comparing neurodevelopment at 2 years [52] and at school age [13, 15] have largely focused on extremely preterm children (22–27 weeks GA) and reported no clear reduction in severe neurodevelopmental impairments over time. Consistent with these findings, our results indicate no overall improvement in neurodevelopmental outcomes between VP infants born in the 1980s and those born in the 2000s, despite some differences in separate milestones.

In this study, we used the same 5 neurodevelopmental milestones to assess fine and gross motor development, as well as communication, in VP infants at 2 years of corrected age. Table 5 provides an overview about the pass rates for these selected milestones in the POPS and LOLLIPOP cohorts (children born 25–31 GA), as well as in full-term (FT) children in the LOLLIPOP cohort [57] and in children from the general population [31]. At 24 months of corrected age, we found severe neurodevelopmental impairment in 1.6% of the infants in the LOLLIPOP cohort and 6.8% in the POPS cohort. Additionally, there was a significant difference in the pass rates for two specific milestones: “imitates others” and “squats or bends to pick things up”, with higher proportion of LOLLIPOP children passing these milestones compared to POPS children. Importantly, the gap between VP infants and the general population that existed in the 1980s, particularly for these two milestones, had narrowed in the 2000s, with VP LOLLIPOP children achieving these milestones at a rate similar to FT children [31, 57].

**Table 5:** Pass rates on 5 selected neurodevelopmental milestones at 24 months of age within our study and other studies conducted with full-term born children or children from the general population

Outcome	Schlesinger Was (1981)^1^	POPS (1983) Very preterms	SMOCK (1988-89)^1^	LOLLIPOP (2022-03) Very preterms	LOLLIPOP (2022-03) Full-terms^2^	Deusloo (2011-13)^1^
Builds tower of three cubes	92/92	90.8	92/90	88.8	91.0	93.1/91.4
Imitates others	96	**94.8**	99	**97.9**	99.0	99.4
Says 'sentences' of two words	65	75.7	83	73.6	86.9	84.8
Squats or bends to pick things up	93	**89.1**	98	**96.0**	96.7	98.1
Walks smoothly	-	90.0	99	91.6	96.9	99.5

Bold: results from the present study

^1^ Deurloo et al., 2021 [31]

^2^Van Dokkum, Bos et al., 2020 [57]

We observed that 24–26% of all VP children involved in our study failed the milestone “says ‘sentences’ of two words” compared to the 15–17% failure rates in the general population in both the 1980s and 2000s [31, 57]. The delay in communication of VP infants at 24 months is particularly striking, as several studies have shown that early vocabulary knowledge is a strong predictor of later language and cognitive development [58–60].

Moreover, 8–10% of VP children failed the milestone ‘walks well alone’, compared to 0–3% of FT children. This confirms the gross motor delay in the VP group, as reported by other authors [61, 62]. However [61], noted that only children born before 30 weeks of gestation showed significant gross motor delays, compared to FT children, while those born at more advanced gestational ages did not have a high rate of gross motor delay. Walking is a key milestone in early motor development, and delays in walking are associated with further gross motor and social impairments at preschool age [63] as well as poorer cognitive performance in adulthood [64].

Early neurodevelopment, including specific milestones, is a valid predictor of developmental delay at school entry [65], school performance during adolescence, and educational achievement in adulthood [66].

Our study, based on two national Dutch cohorts spanning the pre- and post-surfactant eras, therefore contributes to understanding long-term growth and developmental trajectories beyond survival and early neonatal health. The cohorts studied were born several decades ago and neonatal care has changed substantially since then. These changes therefore indicate limited direct generalisation to contemporary cohorts. However, previous research suggests that although neonatal survival and some morbidity outcomes improved by the 1990s [9], since then, this trend appears to have slowed, plateaued, or even reversed in some contexts by the 2020s [67]. Between the 1980s and 2000s, mortality rates among VP infants clearly declined, and some improvements in morbidity were observed in the Netherlands [16]. Additionally, there is no evidence of improvement in cognitive outcomes in very preterm children between the 1970s and 2010s [54].

### Strengths and limitations

This study benefits from the use of two population-based cohorts, covering 94% of the VP population from 1983 to 25% of the preterm population from 2002 to 03 in the Netherlands. This comprehensive dataset provides a more nuanced picture than data from single-centre or multi-centre studies. The Dutch healthcare system offers a unique advantage in monitoring the development of children in their early years, with most costs covered by the government and a large number of physicians involved in preventive healthcare [68]. Children are regularly invited to PCHCs for growth and developmental check-ups using standardized measures such as DDI.

However, both the POPS and LOLLIPOP cohorts were not originally designed for comparative analysis. For this purpose, we conducted a harmonization process between both cohorts, aligning the study populations, and the outcome variables such as growth, neurodevelopment and perinatal variables. Our analysis focused on a selected and well-defined group of infants born at 25–31 weeks of gestation, who survived until 2 years of age without any major congenital malformations. Although both cohorts used the same neurodevelopmental measure, the structure of DDI has changed over time. To ensure a valid comparison, we selected only the milestones from the DDI that were assessed identically in both cohorts.

The selection of cohort participants and DDI milestones presents both strengths and limitations. The chosen sample from both cohorts represents a subgroup of the VP population, primarily those in better health. Additionally, we analysed only five neurodevelopmental milestones between the two cohorts at 24 months of corrected age. The limited number of harmonized milestones means that that we could only assess 1–2 milestones in the domains of fine motor, gross motor and communication. The POPS and LOLLIPOP cohorts assessed slightly different DDI milestones in the first two years of life, and neurodevelopmental milestones were measured at different time points with corrected age in POPS and calendar age in LOLLIPOP. Due to these differences, we limited our comparison to neurodevelopment at 24 months corrected age. A more complex analysis using GA-adjusted z-score trajectories in neurodevelopment could have provided a more comprehensive understanding of changes in VP infants’ neurodevelopment in the first two years of life across eras. We performed all growth and neurodevelopmental calculations using standardised values, as we were not able to compare our own cohort data with matched FT controls.

During the harmonization process, some important health and sociodemographic variables had to be excluded because different definitions were used across the two cohorts. BPD and ethnicity are examples of factors that could potentially explain changes in growth and neurodevelopment over time.

### Implications for further research

Several long-term studies have tracked growth, cognitive and motor development in VP and EP cohorts from birth into adulthood [69–71]. To complement existing evidence and explore new patterns of growth and neurodevelopment in VP children throughout childhood, we strongly encourage to conduct or continue further prospective studies of cohorts born after 2000. Comparison analyses of growth and developmental data between older (1980s/1990s) and newer (2000/2010s) VP cohorts could yield valuable insight into how physical, and neurodevelopment has changed over time. Such investigations should account for both medical (e.g. neonatal) and sociodemographic (e.g. socioeconomic) confounders influencing development.

Many studies report that cognitive, motor and behavioural problems in EP children at school age have not changed across different eras [13, 15]. Furthermore, even mild developmental problems in infancy can become more severe by school age if tailored intervention are not provided [62, 72]. Thus, we recommend rigorous monitoring of infants and preschool-aged children, with early intervention, when necessary, to mitigate the risk of long-term developmental impairments.

## Conclusion

The findings of the present study suggest that height and weight trajectories in the first two years of life have improved for VP infants from the 1980s to the 2000s. The decrease in severe height and neurodevelopmental impairment at 24 months of age in the 2000s cohort can likely be attributed to both sociodemographic and neonatal background factors. However, no improvement in cognitive or motoric functions has been observed across these eras. Given the persistence of developmental challenges, interventions for children born before 32 weeks of gestation should be implemented early, particularly in infancy and in the presence of neonatal morbidities, to support optimal development.

## Supplementary Information

Supplementary Material 1

## Data Availability

Data requests can be submitted to the cohort coordinators (POPS: PD; LOLLIPOP: AFB) who will evaluate this request. Metadata of the POPS cohort data are also available on the RECAP data platform (https://recap-preterm.inesctec.pt/cat). R syntax requests for the current analysis can be submitted to paula.vandommelen@tno.nl.

## Footnotes

1
*cohort harmonization was carried out as described in Sexty et al.*,* 2023 (16)*

2
*chronological age interval: 0*,* 4–27 months for height/weight*,* and 0*,* 4–15 months for head circumference*.

3
*corrected age: 24 months*.

## Abbreviations

BPD: Bronchopulmonary dysplasia
BW: Birth weight
GA: Gestational age
IVH: Intraventricular haemorrhage
LOLLIPOP: Longitudinal Preterm Outcome Project
NICU: Neonatal intensive care unit
OR: Odds ratio
PCHC: Preventive child healthcare centre
POPS: Project on Preterm and Small-for-gestational age infants
VP: Very preterm
