# Supplementary material for: Comparison of early growth and development in very preterm children in the Netherlands between the 1980s and 2000s
Source: BMC Pediatr. 2026 Apr 29;26:399. doi: 10.1186/s12887-026-06857-9 (PMC13134155; doi:10.1186/s12887-026-06857-9)
Supplement: Supplementary file 1 — Supplementary Material 1 [file 12887_2026_6857_MOESM1_ESM.docx]

Suppl. Table 1 Numbers of children attending measurements and all numbers of measurements in POPS and LOLLIPOP

|  | **POPS** | **LOLLIPOP** |
| --- | --- | --- |
| Total number of children | 679 | 515 |
| Number of children with height measurements (4-27 months of age) | 653 | 511 |
| Number of height measurements (4-27 months of age) | 2,160 | 3,916 |
| Number of children with weight measurements (4-27 months of age) | 659 | 512 |
| Number of weight measurements (4-27 months of age) | 2,290 | 4096 |
| Number of children with head circumference measurements (4-15 months of age) | 623 | 500 |
| Number of head circumference measurements (4-15 months of age) | 1,622 | 2,577 |
| Number of children with Developmental score (~24 months of postmenstrual age) | 572 | 373 |
